# Health status of aged women with or without the experience of practicing yoga

**DOI:** 10.1186/s12905-023-02586-8

**Published:** 2023-10-04

**Authors:** Sarah Suet Shan Wong, Tai Wa Liu, Shamay Sheung Mei Ng

**Affiliations:** 1 Department of Rehabilitation Sciences, The Hong Kong Polytechnic University, Hung Hom, Hong Kong (SAR), China; 2 School of Nursing and Health Studies, Hong Kong Metropolitan University, Ho Man Tin, Hong Kong (SAR) China; 3 Research Centre for Chinese Medicine Innovation, The Hong Kong Polytechnic University, Hong Kong (SAR), Hung Hom, Hong Kong (SAR), China

**Keywords:** Yoga, Health promotion, Physical activity, Wellness

## Abstract

**Background:**

Yoga is a popular training practice that enhances women’s physical activity level and modifies the major risk factors contributing to noncommunicable diseases. This study aimed to compare general health and cardiovascular health, musculoskeletal health, psychological health, and health-related quality of life between aged women with and without long-term yoga practice.

**Methods:**

Thirty-two female yoga practitioners (mean age 56 years) with ≥ 2 years experience in regular yoga practice and 32 age-matched women without yoga experience participated in the study. Between-group comparisons was performed to explore the differences in various health outcomes, including body build indices, exercise endurance, blood pressure, and heart rate variability; hamstring flexibility, upper-limb muscle strength, shoulder range of motion, and upper-limb function; and the symptoms of anxiety and depression, sleep quality, and fatigue.

**Results:**

Our findings revealed that yoga practitioners demonstrated greater hamstring flexibility, shoulder ROM on the non-dominant side, and hand-grip strength; a higher heart rate variability parameter value (RMSSD); and shorter sleep latency than those who did not practice yoga.

**Conclusions:**

In view of the encouraging results of the long-term benefits of yoga practice, it warrants being promoted among aged women to enhance their physical and mental well-being.

**Supplementary Information:**

The online version contains supplementary material available at 10.1186/s12905-023-02586-8.

## Background

Yoga is a form of integrative medicine that the mind, body, and spirit are unitive in nature [1]. While various aspects of physical fitness, including limb flexibility, balance, and muscle strength, can be improved by the physical postures and breathing exercises used during the practice of yoga, the meditation component has been shown to improve mood. The lifetime prevalence rate of practicing yoga is as high as 13.2% in the general population in the US (N = 34,525) [2], and this trend has been increasing, particularly among older-aged women with better-rated general health [3]. Further, female yoga practitioners have a more positive body image and higher levels of self-esteem than women who do not practice yoga [4]. The practice of yoga can provide a supportive way to promote physical and psychological health, prevent general diseases, and alleviate chronic medical conditions.

Physical inactivity is one of the major risk factors contributing to noncommunicable diseases (NCDs), including cardiovascular disease, stroke, diabetes, and cancer, which lead to subsequent economic burdens and mortality [5]. Based on the World Health Organization’s (WHO) investigation, inactivity increases with age, and more women (33.9%) than men (27.9%) are reported to be less physically active in different countries [6]. To achieve the WHO’s established goals of reducing physical inactivity by 10% and 15% by 2025 and 2030, respectively [7], it is important to address these age and gender gaps in physical inactivity.

Recent studies have reported the potential benefits of yoga in improving women’s health. For example, Wang et al. [8] systematically meta-analyzed 19 randomized controlled trials involving 1,832 women with sleep problems and revealed that yoga therapy significantly improved sleep compared to control participants (standardized mean difference [SMD] = − 0.327; 95% confidence interval [CI] = − 0.506, − 0.148). In another study, Forseth et al. [9] compared 74 yoga practitioners, of which 80% were female and had a mean age of 41.3 ± 1.5 years, with physically inactive participants (n = 1,285; 43.8% female, mean age = 46.4 ± 0.4 years) and found that yoga practitioners were less likely to have an elevated waist circumference (odds ratio [OR] = 0.30; *p* < 0.01), body mass index (OR = 0.48; *p* = 0.047), and triglyceride concentration (OR = 0.34; *p* = 0.02). Moreover, Satin et al. [10] compared 47 yoga practitioners (57% female) who had a mean age of 39.51 ± 11.02 years, with physically inactive participants (n = 52; 54% female, mean age = 34.58 ± 12.55 years), and found that the yoga practitioners had lower levels of depressed mood (Cohen’s d (*d*) = − 0.71; *p* = 0.001) and perceived stress (*d* = 0.88; *p* < 0.001).

Although recent literature [8–10] compared the potential benefits of practicing yoga, they only focused on the effects of those with young to middle age and short period of practicing yoga. For example, in Forseth et al. [9] and Satin et al. [10] studies, the included participants were women with young to middle age (mean age < 45 years); in Cramer et al. [11] and Elwy et al. [12] studies, the included participants were with only 4 to 16 weeks experience of yoga practice. Moreover, abundant studies focused on disease populations, such as cardiovascular diseases [13], pulmonary diseases [14], cancer [15], musculoskeletal disorders [16], diabetes [17], but the potential effects of yoga on healthy women were understudied. Therefore, to understand the effects of long-term yoga practice on health variables in relation to its physiological resilience and homeostatic capacity, the objectives of this study were to compare the general health, cardiovascular health, musculoskeletal health, psychological health, and health-related quality of life between women with and without the experience of long-term yoga practice.

## Materials and methods

### Study Design and participants

This cross-sectional study was conducted at a university-affiliated rehabilitation laboratory in Hong Kong from April to September 2022. Participants were recruited through electronic postings on social media platforms and poster advertisements at yoga associations/studios. Yoga practitioners were included if they: (1) were women aged 45 to 75 and (2) self-reported regularly engaging in any form of yoga for more than 2 years, with at least 60 min weekly. Yoga practitioners were excluded if they: (1) were diagnosed with a major medical condition that may have hindered their proper assessment, e.g., cardiovascular, pulmonary, neurological, cognitive, or psychological disorders or (2) had prior experiences of practicing stretching exercise or meditation other than yoga in the past 6 months. For the non-yoga group, the inclusion and exclusion criteria were the same as the yoga group, except that they did not have any experience with yoga practice.

### Setting and procedure

The eligible participants were invited to the Wellness & Exercise Laboratory at the administering university for data collection. After obtaining data regarding sociodemographics and the physical activity level, which was assessed using the Chinese version of the International Physical Activity Questionnaire (IPAQ) [18], all assessments were then administered in a random order by two research assistants who were trained by an experienced research nurse. At least 5 min of rest were allowed between tests to avoid fatigue. The study procedure was explained to each participant, and written informed consent was obtained prior to commencing the study. The study protocol was approved by the institutional review board of the administering university (reference number: HSEARS20220202003) and was conducted in accordance with the guidelines for human experiments outlined in the Declaration of Helsinki.

### Outcome measures

#### Assessments of general and cardiovascular health

The body mass index (BMI) and waist-to-height ratio (WHtR) were measured as a proxy for obesity. The participants’ body weight, height, and waist circumference were measured to calculate body build indices.

The 6-Minute Walk Test (6MWT) [19], which measures the maximum walking distance achieved in 6 min, reliably determines exercise endurance. Heart rate and oxygen saturation were measured before and immediately after the test.

Blood pressure, heart rate, and heart rate variability (HRV) were recorded to determine cardiovascular function. HRV, which was used as a measure of autonomic nervous system function [20], was recorded over a 5-minute period using a validated wearable monitor (Polar H10 heart rate sensor, POLAR®, Kempele, Finland) [21]. Time-domain measures, including the mean R-R interval (RRi); the standard deviation of normal-to-normal intervals (SDNN); the square root of the mean squared differences of successive normal-to-normal intervals (RMSSD); the proportion of NN50 divided by total number of NN intervals (pNN50); and frequency-domain measures, including total power, low frequency normalized units (LF n.u.), high frequency normalized units (HF n.u.), and the LF/HF ratio, were recorded [22]. The analysis was supported by Kubios software [23].

#### Assessments of musculoskeletal health

The Sit-and-Reach Test was used to assess the extensibility of the lower back and hamstring [24]. The participants were seated with their knees fully extended and one hand on top of the other. They then attempted to stretch forward as far as possible. The distance from the tip of the middle finger to the toe was measured, and the best score was recorded from three attempts.

Shoulder mobility was determined by measuring the active range of motion (ROM) of the bilateral shoulder joints in five angles (flexion, extension, abduction; internal rotation [IR] and external rotation [ER] with the shoulder at 90° abduction), using a goniometer [25]. In addition, the hand-behind-back (HBB) ROM was measured by measuring the interval starting from cervical seventh (C7) vertebra to the level that participant’s thumb maximally reached along the spinal column [26]. Hand-grip strength was measured using a handheld dynamometer (Baseline® Smedley Spring, USA), and shoulder flexion and abduction muscle strength were determined as the strength of a maximal voluntary isometric contraction measured using a handheld dynamometer (Model 001165; Lafayette Instrument, Lafayette, IN, USA). Satisfactory intra-tester reliability has been demonstrated for testing the shoulder ROM (intraclass correlation coefficient [ICC]_3,1_ = 0.84–1.0) and muscle strength (ICC_3,1_ = 0.72–0.99) [25]. The average of three measurements taken in each position was used for analysis.

The upper limb functionality was assessed using the short form of the Chinese (Hong Kong) version of the Disabilities of Arm–Shoulder–Hand Questionnaire (quickDASH-HKPWH) [27]. The quickDASH contains 11 items (0–100%; 0 = no disability; 100 = most severe disability). It has demonstrated good internal consistency (Cronbach’s alpha = 0.94) and test–retest reliability (ICC = 0.77) [27].

#### Assessments of psychological health

The participants’ anxiety and depressive symptoms were assessed using the Chinese version of the Hospital Anxiety and Depression Scale (HADS-Chinese), which has demonstrated good internal consistency (Cronbach’s alpha = 0.85) [28]. The anxiety (HADS-A) and depression (HADS-D) subscale scores range from 0 to 21, with a higher score indicating more severe anxiety and depression.

Sleep quality was assessed using the Chinese version of the Pittsburgh Sleep Quality Index (PSQI-C), which has demonstrated good reliability [29]. The PSQI-C has 19 items (score: 0–21) consisting of the following seven components: subjective sleep quality, sleep latency, sleep duration, sleep efficiency, sleep disturbance, the use of sleep medication, and daytime dysfunction. A higher score represents poorer sleep.

Fatigue was assessed using the Chinese (Cantonese) version of the Fatigue Assessment Scale (C-FAS), which has demonstrated satisfactory internal consistency (Cronbach’s alpha = 0.71–0.82) and test–retest reliability (ICC = 0.77–0.95) [30]. The C-FAS comprises 10 items (score: 10–50) assessing physical and mental fatigue, with a higher score representing greater fatigue severity.

#### Health-related quality of life

The Chinese (Hong Kong) version of the Short Form 12-Item Health Survey version 2 has demonstrated good reliability and was used to measure health-related quality of life^31^. The mental component score (MCS) and physical component score (PCS) range from 0 to 100, with a higher score indicating a better health-related quality of life .

### Statistical analyses

Statistical Package for the Social Sciences (version 26) software (IBM, Armonk, NY, USA) was used to perform the statistical analyses. Descriptive statistics were used to summarize the demographic characteristics and variables of interest. The significance level in all tests was set as *p* < 0.05. The normality of the data distribution was checked using the Kolmogorov–Smirnov test. For between-group comparisons of continuous variables, independent Student’s *t*-tests and Mann–Whitney *U* tests were used for normally and non-normally distributed data, respectively. For categorical variables, chi-square tests were used. Bivariate analyses of the correlation between yoga practice (e.g. duration of yoga experience) and the outcome variables were performed using Pearson’s product-moment or Spearman’s correlation coefficients for normally and non-normally distributed data, respectively.

## Results

### Participant characteristics

Thirty-two yoga practitioners with a mean age of 56.00 ± 8.68 years and a mean duration of yoga experience of 101.91 ± 56.41 months (range: 2–17 years) and 32 age-matched non-yoga practitioners participated in the study. There were no significant differences in sociodemographic data between the groups (Table 1). Apart from regular yoga practice, both group participants did not regularly engage in other form of physical exercise. Half of the yoga practitioners had practiced yoga for ≥ 10 years (Table 2). All yoga practitioners had engaged in two forms of yoga, hatha yoga and flow yoga, which include components of breathing exercises, yoga posture, and meditation.

**Table 1:** Participants’ characteristics

Demographics	Yoga group (n = 32)	Non-yoga group (n = 32)	Test (*t* or χ2), *p*-value
Age (years), mean (SD) ^†^	56.00 (8.68) [range:45–75]	56.28 (8.54) [range:45–74]	0.131, 0.896
Education, n (%) ^#^			3.135, 0.824
Primary level or below	0	2 (6.2)	
Secondary level	17 (53.1)	12 (37.5)	
Tertiary level	15 (46.9)	18 (56.3)	
Living status, n (%) ^#^			4.010, 0.104
Living alone	6 (18.8)	1 (3.1)	
Living with family	26 (81.3)	31 (96.9)	
Employment, n (%) ^#^			3.590, 0.309
Full time	13 (40.6)	18 (56.3)	
Part time	5 (15.6)	7 (21.9)	
Housewife	3 (9.4)	2 (6.3)	
Retired	11 (34.4)	5 (15.6)	
Marital status, n (%) ^#^			3.221, 0.359
Single	13 (40.6)	7 (21.9)	
Married	17 (53.1)	21 (65.6)	
Divorced	1 (3.1)	3 (9.4)	
Widowed	1 (3.1)	1 (3.1)	
Past medical history, n (%) ^#^			0.563, 0.453
Good past health	18 (56.3)	15 (46.9)	
Medical history	14 (43.8)	17 (53.1)	
Hypertension	4	8	
Hyperlipidemia	6	7	
Diabetics	2	1	
Thyroid disease	1		
Cardiovascular disease	1	1	
IPAQ level, n (%) ^#^			0.110, 1.00
High	6 (18.7)	5 (15.6)	
Moderate	26 (81.3)	27 (84.4)	
Low	0	0	

^†^Independent *t* test; ^#^Chi square test

**Table 2:** Frequency of yoga practice among yoga participants

Yoga practice	Yoga group (n = 32)
Duration of yoga experience (month), mean (SD)	101.91 (56.41)
> 2 years & <10 years, n (%)	16 (50)
> 10 years, n (%)	16 (50)
Yoga practice frequency per week, mean (SD)	1.53 (0.57)
Once a week, n (%)	16 (50)
Twice a week, n (%)	15 (46.9)
Three times a week, n (%)	1 (3.1)
Yoga practice per session (minute), mean (SD)	61.88 (7.38)
60 min, n (%)	30 (93.8)
90 min, n (%)	2 (6.2)
Yoga practice per week (minute), mean (SD)	95.63 (39.91)
60 min, n (%)	16 (50)
120 min, n (%)	13 (40.6)
180 min, n (%)	3 (9.4)

### Between-group differences in outcome measures

#### General and cardiovascular health

There were no significant between-group differences in body build indices or 6MWT results (Appendix I Table 1). Yoga practitioners had significantly higher RMSSD value (50.6%) and HF n.u. (71.4%) and significantly lower LF n.u. (24.1%), and LF/HF ratio (65.3%) than the non-yoga practitioners (*p* < 0.001 to *p* = 0.015).

#### Musculoskeletal health

In upper-limb flexibility assessments, the yoga practitioners had significantly longer sit-and-reach distances (89.9%; *p* = 0.014) and better shoulder ROM in angles of extension (non-dominant side [NDS], 15.2%), internal rotation (NDS, 15.1%), external rotation (NDS, 9.8%; dominant side (NS), 8.9%) and hand-behind-back (NDS, 22.4%) than the non-yoga-practitioners (*p* < 0.001 to *p* = 0.008). Regarding muscle strength, the yoga practitioners had significantly greater body-weight-adjusted hand-grip strength in both the dominant side (12.8%) and non-dominant side (11.7%) and shoulder flexion in the non-dominant side (13.2%) than the non-yoga-practitioners (*p* = 0.007 to *p* = 0.02). However, there were no significant between-group differences in quickDASH scores.

#### Psychological health and quality of life

Among the sleep parameters assessed, the yoga practitioners had a significantly shorter PSQI-latency time (46.1%) than the non-yoga-practitioners (*p* = 0.033). However, there were no significant between-group differences in FAS, HADS-A, HADS-D, or SF12v2 scores.

### Correlations

The correlation analysis showed that the duration of the yoga experience was significantly correlated with the waist-to-height ratio (*r* = −0.385, *p* = 0.031). There were no significant correlations between yoga practice and other outcome variables.

## Discussion

This study is the first to comprehensively report differences in general and cardiovascular health, musculoskeletal health, and psychological health between middle-aged women with and without yoga practice. Our findings revealed that female yoga practitioners demonstrated greater hamstring flexibility, shoulder ROM on the non-dominant side, and hand-grip strength; higher HRV (RMSSD); and shorter sleep latency than non-yoga-practitioners. In addition, a longer yoga experience was associated with a lower waist-to-height ratio.

### General health

Despite no significant between-group differences in BMI or WHtR, the yoga practitioners’ body build indices were within the normal range, whereas the non-yoga-practitioners, with a BMI of 23.23 and a WHtR of 0.5, were classified as overweight [32, 33]. Yoga exercise, which is categorized as low-to-moderate intensity training, may not induce sufficient stress to the cardiovascular system. This may explain the lack of a significant difference in body composition between the yoga practitioners and non-yoga-practitioners. In contrast, Lauche et al. [34] meta-analyzed 30 studies involving 2,173 members of the general population and found that, compared with usual care, a yoga intervention significantly reduced the BMI (SMD = -0.99, *p* = 0.004) of overweight or obese participants, although there were no significant effects on weight-related outcomes in healthy adults. Further, Neumark-Sztainer et al. [35] performed a 5-year longitudinal analysis and showed that weight control could be attained when yoga was performed regularly. Likewise, our analysis showed that a longer yoga experience was associated with reduced WHtR among yoga practitioners (*r* = −0.385). This may be explained by the proficiency in posture of experienced yoga practitioners, who have a higher level of muscle engagement during their practice and therefore increase their energy expenditure and change their body composition [36].

### Cardiovascular health

Our results showed that both groups exhibited similar normative values of systolic blood pressure and distance covered in the 6MWT for healthy women. Inconsistent findings regarding the effect of yoga on exercise endurance have been reported in the literature. For example, Widjaja et al. [37] reported significantly improved 6MWT (6%) by obese older women after a yoga intervention with dynamic and static postures training, whilst McCaffrey et al. [38] found no significant improvement in the 6MWT after seated yoga among elderly individuals with Alzheimer’s disease. As noted, the intensity of physical activity depends on the yoga style. Specifically, Cowen and Adams [39] indicated that a vigorous yoga styles with dynamic movements and strength may enhance physical fitness in comparison to gentle styles focusing on stretching and meditation. The yoga practitioners in our study engaged in relatively gentle styles compared to other forms such as Bikram, ashtanga, and vinyasa. Moreover, effects of exercise on cardiorespiratory fitness could be modified by age, sex, and health status; and men with younger age (< 50 years) and chronic health conditions, such as type 2 diabetes and hypertension, could be better benefited from exercise training [40]. However, our included participants were healthy women with older age (mean age 56.00 ± 8.68). Thus, the effects of yoga on cardiorespiratory fitness were not prominent and not reflected by the 6MWT. Further, our findings were in contrast to those of recent meta-analyses targeting patients with pulmonary disease and hypertension, in which a yoga intervention was found to significantly improve 6MWT (mean difference [MD] = 25.53 m; 95% CI = 12.16, 38.90) [41] and lower systolic blood pressure (MD = -4.17; 95% CI = -6.35, -1.99) [42] when compared with usual care. These previous studies included participants with chronic diseases, whereas our study included healthy participants. Therefore, the findings are not comparable and further investigation is warranted to understand the effects of yoga on cardiopulmonary function.

### Musculoskeletal health

Our positive findings regarding flexibility and strength showed that the yoga practitioners had greater shoulder ROM and hamstring flexibility, as well as body-weight-adjusted hand-grip strength, than the non-yoga-practitioners. The between-group differences in non-dominant shoulder mobility may be due to the non-dominant side being relatively less involved in daily activities, whereas yoga improves its flexibility. The repetition of the tension and relaxation of limb muscles during yoga posture improves flexibility, which is further enhanced with synchronized breathing practice. Yoga training on posture, particularly training involves weight supporting with upper limbs, is thought to be conducive to improving muscle strength. Yoga has been shown to improve hand-grip strength among teens and young adults (8.9-27.3%) [43] and older females (16.6%) [44], but it has not been investigated in middle-aged people. In addition to the effects of posture training, another possible cause of the improvement in hand-grip strength may be the voluntarily controlled yogic breathing exercise, although the associated mechanism is not well established. It is hypothesized that yogic breathing increases cardiac autonomic balance and parasympathetic tone, which have been found to be associated with a reduction in oxygen consumption for work by the body, thereby providing more energy to increase grip strength [45].

### Psychological health

In our study, the regulation of autonomic balance among yoga practitioners was indicated by the findings of HRV parameters, which reflected the predominance of parasympathetic tone coupled with the withdrawal of sympathetic tone in yoga practitioners. The effect of yoga on HRV is inconclusive, with preliminary findings suggesting that regular yoga practice increases HRV and vagal tone [46]. It may be affected independently by yogic breathing. Together with the calming effect of meditation and the use of pose practice to stimulate peripheral pressure receptors innervated by the parasympathetic afferent route [47], yoga has been suggested to regulate cardiac autonomic nervous modulation.

Although we found no between-group differences in HADS scores, the proportion of borderline to abnormal levels of anxiety was two-fold higher in the non-yoga-practitioners (28.2%) than the yoga practitioners (12.5%). Further, a shorter sleep latency was found in the yoga practitioners relative to the non-yoga-practitioners. Prolonged stress and anxiety lead to dysfunctional arousal, which induces difficulties in falling asleep, therefore, lengthened sleep latency.

Mood states are affected by various factors, such as the environmental context and our cognition. From the physiological aspects, they are associated with the autonomic nervous system activity and HRV is a measure of the parasympathetic and sympathetic nervous systems. In the present study, we found that yoga practitioners had higher HF and RMSSD and lower LF and HF/LF ratio than non-yoga-practitioners. These findings were consistent with previous studies [48, 49] that women with yoga practicing experience could have increased parasympathetic tone and decreased sympathetic tone that may lead to the mobilization of our stress coping capacity. This may explain that our yoga practitioners had lower level of anxiety as measured by the HADS than non-yoga-practitioners.

Further, brain imaging findings indicate that experienced yoga practitioners have morphological changes in functional regions and hippocampal circuits of the brain [50] that are associated with emotional regulation, including anxiety reduction. The breathing control training and meditation components of yoga practice may help maintain homeostasis to regulate the circadian sleep–wake cycle, which can alter sleep onset and thereby improve sleep latency. It is believed that the physical component of yoga improves sleep, but breathing control training and meditation components also make contribution.

Previous studies revealed that yoga has small effects (SMD = 0.27, 95% CI = 0.23, 0.31) [51] on improving the fatigue severity with various physical diseases, such as cancer, but it was not reflected in this cohort of participants with at least 2 years of yoga experience when compared with those without yoga practicing experience. One possible explanation is that, in the present study, our participants were healthy subjects with low level of fatigue (mean FAS score < 22). Thus, the effects of yoga could not further induce mark improvement of fatigue. Another possible explanation is that is our sample size (yoga group, n = 32) was not sufficient to capture the small effects of yoga on improving fatigue.

### Quality of life

Surprisingly, our results showed no significant difference in health-related quality of life between yoga group and non-yoga group. This is in contrast to previous studies that proposed that practicing yoga improves quality of life through several possible mechanisms, including improved physical fitness, neurocardiac modulation, cognitive skills, and emotions through yoga poses, breathing control training, and meditation, as well as improved social participation [52, 53]. A possible reason for the discrepancies is that those participants may suffer from symptoms of chronic disease, thus, they may have benefited more from a yoga intervention. Despite our postulation that yoga practitioners have a better quality of life than non-yoga-practitioners, the healthy participants of our study may have had a relatively high health-related quality of life (SF12v2: 50.14–51.57), and thus no significant between-group differences were observed. Moreover, although the non-yoga group did not engage in yoga practice, they may have engaged in other activities that may have also induced a better quality of life.

## Limitations

There are several limitations of this study. First, there was a lack of detailed information on the yoga styles adopted. An analysis of yoga type would help to understand how yoga practice brings specific health benefits. Second, our participants were recruited on a convenience basis, therefore, self-selection bias cannot be ruled out. Further, our rather homogenous subject group may limit the generalizability of the findings. Third, the small sample size potentially influenced the statistical power to expose the effects on certain outcomes. Finally, due to the cross-sectional design of the study, a cause-and-effect relationship between yoga and health outcomes could not be established. Future research directions should include an evaluation of the effects of yoga on various health outcomes in large-scale randomized controlled trials.

## Conclusions

Our findings revealed that aged women with long-term yoga practice outperformed their non-yoga-practicing counterparts in hand-grip strength, shoulder flexibility, particularly non-dominant hand mobility, cardiac autonomic function, and sleep latency. In view of the benefits of this multimodal activity, it is worth promoting yoga among aged women to enhance their physical and mental well-being.

### Electronic supplementary material

Below is the link to the electronic supplementary material.

Supplementary Material 1

## Data Availability

The datasets used and/or analysed during the current study are available from the corresponding author on reasonable request.

## Abbreviations

IPAQ: International Physical Activity Questionnaire
BMI: Body mass index
WHtR: Weight-to-height ratio
HBB: hand-behind-back
HR: heart rate
6MWT: 6-min walk test
HRV: heart rate variability
RRi: R-R interval
SDNN: standard deviation of normal-to-normal intervals
RMSSD: square root of the mean squared differences of successive normal-to-normal intervals
pNN50: proportion of NN50 divided by total number of NN intervals
LF n.u.: low frequency normalized unit
HF n.u.: high frequency normalized unit
quickDASH: short form of the Disabilities of Arm-Shoulder-Hand Questionnaire
PSQI: Pittsburgh Sleep Quality Index
HADS: Hospital Anxiety and Depression Scale
FAS: Fatigue Assessment Scale
SF-12v2: Short Form 12-item Health Survey version 2
PCS: physical component score
MCS: mental component score
