# Supplementary material for: Health status of aged women with or without the experience of practicing yoga
Source: BMC Womens Health. 2023 Oct 4;23:524. doi: 10.1186/s12905-023-02586-8 (PMC10552255; doi:10.1186/s12905-023-02586-8)
Supplement: Supplementary file 1 — Supplementary Material 1 [file 12905_2023_2586_MOESM1_ESM.docx]

**Appendix I**

**Table 1: Comparison of variables of interest**

|  | Yoga group (n=32)  mean (SD) | Non-yoga group (n=32)  mean (SD) | Test (t, U or χ2),  *p*-value |
| --- | --- | --- | --- |
| **General health** |  | | |
| body mass index (kg/m^2^)^✝^ | 21.96 (2.57) | 23.32 (2.92) | 1.988, 0.051 |
| waist-height ratio (WHtR)^✝^ | 0.48 (0.06) | 0.50 (0.06) | 1.610, 0.113 |
| **Cardiovascular health** |  | | |
| systolic BP (mmHg) ^✝^ | 125.00 (13.71) | 129.38 (12.98) | 1.311, 0.195 |
| diastolic BP (mmHg) ^✝^ | 78.09 (7.80) | 80.75 (9.16) | 1.249, 0.217 |
| resting HR (bpm) ^✝^ | 76.31 (10.48) | 76.88 (9.97) | 0.220, 0.827 |
| **exercise endurance** |  |  |  |
| 6MWT (distance, m) ^✝^ | 553.28 (67.72) | 546.78 (83.87) | -0.341, 0.734 |
| pre HR (bpm) ^✝^ | 73.53 (10.25) | 76.66 (9.94) | 1.272, 0.208 |
| post HR (bpm) ^✝^ | 114.84 (15.66) | 117.31 (13.15) | 0.683, 0.497 |
| pre SpO2 (%)^✝^ | 98.50 (0.51) | 98.69 (0.47) | 1.531, 0.131 |
| post SpO2 (%)^✝^ | 97.56 (0.95) | 97.34 (1.00) | 0.814, 0.374 |
| **heart rate variability** |  |  |  |
| min heart rate (bpm) ^✝^ | 68.50 (8.55) | 71.56 (9.58) | 1.349, 0.182 |
| max heart rate (bpm) ^✝^ | 87.97 (16.29) | 88.75 (10.37) | 0.238, 0.813 |
| mean heart rate (bpm) ^✝^ | 75.63 (9.99) | 78.41 (10.54) | 1.083, 0.283 |
| mean RRi (ms) ^✝^ | 808.13 (110.88) | 778.470 (104.35) | -1.102, 0.275 |
| SDNN (ms) ^✝^ | 29.92 (13.32) | 24.92 (10.65) | -1.660, 0.102 |
| RMSSD (ms) ^✝^ | 35.51 (20.85) | 23.58 (16.93) | -2.513, 0.015* |
| pNN50 (%)^✝^ | 9.43 (11.71) | 5.39 (11.71) | -1.527, 0.132 |
| total Power^✝^ | 768.19 (810.92) | 557.14 (420.98) | -1.307, 0.198 |
| LF n.u. ^✝^ | 49.00 (18.78) | 64.52 (23.00) | 2.958, 0.004** |
| HF n.u. ^✝^ | 51.08 (18.76) | 29.81 (20.88) | -4.286, 0.000** |
| LF/HF ratio^✝^ | 1.34 (1.17) | 3.86 (3.53) | 3.831, 0.000** |
| **Musculoskeletal health** |  | | |
| **hamstring flexibility** |  |  |  |
| sit-and-reach (cm) ^✝^ | 8.43 (5.40) | 4.44 (7.08) | -2.534, 0.014* |
| **shoulder ROM (dominant)** |  | | |
| flexion (°)^ǂ^ | 170.16 (6.16) | 169.89 (8.08) | 491.500, 0.783 |
| abduction (°)^✝^ | 166.06 (10.76) | 167.87 (9.50) | 0.714, 0.478 |
| extension (°)^ǂ^ | 58.77 (7.16) | 62.35 (38.19) | 366.500, 0.051 |
| internal rotation (°)^✝^ | 55.22 (10.87) | 50.40 (10.89) | -1.773, 0.081 |
| external rotation (°)^✝^ | 107.55 (5.72) | 98.76 (6.96) | -5.514, 0.000** |
| HBB (cm) ^ǂ^ | 13.22 (3.91) | 15.28 (4.58) | 375.000, 0.066 |
| **shoulder ROM (non-dominant)** |  | | |
| flexion(°)^✝^ | 171.09 (5.84) | 169.23 (8.89) | -0.993, 0.325 |
| abduction(°)^✝^ | 171.03 (8.31) | 168.56 (9.54) | -1.103, 0.274 |
| extension(°)^✝^ | 65.97 (7.70) | 57.28 (10.72) | -3.723, 0.000** |
| internal rotation(°)^ǂ^ | 58.72 (13.37) | 51.01 (12.47) | 314.000, 0.008** |
| external rotation(°)^ǂ^ | 106.97 (6.57) | 97.45 (8.54) | 216.500, 0.000** |
| HBB (cm) ^✝^ | 10.73 (2.77) | 13.82 (4.10) | 3.540, 0.001** |
| **Body-weight-adjusted**  **muscle strength (dominant)**  **[Muscle strength, kg]** |  | | |
| shoulder flexion^✝^ | 0.456 (0.11)  [9.94±2.39] | 0.418 (0.10)  [9.58±1.93] | -1.421, 0.160 |
| shoulder abduction^✝^ | 0.441 (0.11)  [9.59±2.32] | 0.390 (0.12)  [8.91±2.47] | -1.728, 0.089 |
| hand-grip^✝^ | 1.23 (0.17)  [26.89±4.12] | 1.09 (0.22)  [25.05±3.65] | -2.781, 0.007** |
| **Body-weight-adjusted**  **muscle strength (non-dominant)**  **[Muscle strength, kg]** |  | | |
| shoulder flexion^✝^ | 0.420 (0.09)  [9.16±1.90] | 0.371 (0.10)  [8.51±2.13] | -2.040, 0.046* |
| Shoulder abduction^✝^ | 0.387 (0.08)  [8.41±1.55] | 0.343 (0.11)  [7.85±2.24] | -1.834, 0.072 |
| hand grip^✝^ | 1.15 (0.18)  [25.13±4.06] | 1.03 (0.24)  [23.49±4.26] | -2.391, 0.020* |
| **upper limb function** |  |  |  |
| quickDASH ^ǂ^ | 6.53 (6.58) | 7.74 (9.76) | 504.500, 0.919 |
| quickDASH-work ^ǂ^ | 3.32 (5.71) | 4.69 (6.74) | 462.000, 0.438 |
| quickDASH-sport ^ǂ^ | 9.57 (12.30) | 6.56 (11.00) | 284.000, 0.456 |
| **Psychological health** |  | | |
| **sleep** |  |  |  |
| PSQI global ^ǂ^ | 4.94 (2.71) | 5.97 (3.00) | 428.500, 0.259 |
| poor sleep (PSQI>5)^#^ | n=12 (37.5%) | n=17 (53.1%) | 1.576, 0.209 |
| **Sub-score of PSQI** |  |  |  |
| duration ^ǂ^ | 1.13 (0.61) | 1.16 (0.77) | 504.000, 0.904 |
| disturbance ^ǂ^ | 1.06 (0.44) | 1.16 (0.68) | 464.000, 0.436 |
| latency ^ǂ^ | 0.69 (0.74) | 1.28 (1.11) | 361.500, 0.033* |
| day dysfunction ^ǂ^ | 0.50 (0.62) | 0.69 (0.69) | 438.000, 0.269 |
| quality ^ǂ^ | 1.03 (0.70) | 1.28 (0.77) | 411.500, 0.138 |
| efficiency ^ǂ^ | 0.38 (0.66) | 0.28 (0.46) | 498.500, 0.817 |
| medication ^ǂ^ | 0.13 (0.42) | 0.13 (0.42) | 512.000, 1.000 |
| **Mood** |  |  |  |
| HADS-anxiety^✝^ | 4.06 (2.94) | 5.19 (3.33) | 1.434, 0.157 |
| anxiety level ^ǂ^ |  |  | 432.500, 0.127 |
| normal (0-7) | n=28 (87.5%) | n=23 (71.9%) |  |
| borderline (8-10) | n=3 (9.4%) | n=7 (21.9%) |  |
| abnormal (11-21) | n=1 (3.1%) | n=2 (6.3%) |  |
| HADS-depression^✝^ | 2.88 (2.95) | 3.56 (2.49) | 1.008, 0.317 |
| depression level ^ǂ^ |  |  | 498.500, 0.764 |
| normal (0-7) | n=28 (87.5%) | n=27 (84.4%) |  |
| borderline (8-10) | n=3 (9.4%) | n=5 (15.6%) |  |
| abnormal (11-21) | n=1 (3.1%) | n=0 |  |
| **Fatigue** |  |  |  |
| FAS (total) ^✝^ | 19.97 (4.39) | 21.47 (5.57) | 1.197, 0.236 |
| Fatigue level ^ǂ^ |  |  | 504.000, 0.902 |
| No fatigue | n=16 (50%) | n=17 (53.1%) |  |
| Fatigue | n=16 (50%) | n=14 (43.8%) |  |
| Extreme fatigue | n=0 | n=1 (3.1%) |  |
| **Sub-score of FAS** |  |  |  |
| FAS (physical) ^ǂ^ | 11.06 (3.09) | 12.22 (3.67) | 458.000, 0.465 |
| FAS (mental) ^ǂ^ | 8.91 (2.15) | 9.25 (2.49) | 494.000, 0.807 |
| **Quality of life** |  | | |
| PCS ^ǂ^ | 51.57 (5.83) | 50.56 (8.18) | 509.500, 0.973 |
| MCS^✝^ | 51.32 (7.47) | 50.14 (6.80) | -0.661, 0.511 |
| **Sub-score of SF-12v2** |  |  |  |
| physical functioning ^ǂ^ | 51.89 (6.81) | 51.16 (7.74) | 505.000, 0.917 |
| role physical ^ǂ^ | 51.78 (5.44) | 49.79 (7.78) | 452.000, 0.398 |
| bodily pain ^ǂ^ | 52.00 (6.17) | 51.53 (7.75) | 508.000, 0.953 |
| general health ^ǂ^ | 46.51 (9.63) | 45.45 (11.93) | 497.000, 0.832 |
| vitality ^ǂ^ | 57.67 (6.49) | 55.83 (8.45) | 458.000, 0.428 |
| social functioning ^ǂ^ | 52.45 (5.53) | 51.90 (6.75) | 504.000, 0.903 |
| role emotional ^ǂ^ | 47.37 (7.51) | 46.86 (8.29) | 504.500, 0.918 |
| mental health ^ǂ^ | 51.66 (8.67) | 49.87 (6.99) | 427.500, 0.244 |

^✝^ Independent t test; ^ǂ^ Mann–Whitney U test; ^#^Chi square test; * p<0.05; **p<0.01
